# Bringing Psilocybin-Assisted Therapy to Palliative Oncology: Early Lessons from Real-World Implementation

**DOI:** 10.3390/healthcare14111559

**Published:** 2026-06-03

**Authors:** Michel Dorval, Virginie Audet-Croteau, Sue-Ling Chang, Marianne Masse-Grenier, Annie Tremblay, Elodie Bénard, Alexandra Chapdelaine, Nicolas Garel, Jason Robert Guertin

**Affiliations:** 1 Faculty of Pharmacy, Université Laval, Quebec, QC G1V 0A6, Canada; 2 Oncology Program, CHU de Québec-Université Laval Research Center, Quebec, QC G1S 4L8, Canada; virginie.audet-croteau.med@ssss.gouv.qc.ca (V.A.-C.); sue-ling.chang@crchudequebec.ulaval.ca (S.-L.C.); marianne.masse-grenier@crchudequebec.ulaval.ca (M.M.-G.); annie.tremblay.med@ssss.gouv.qc.ca (A.T.); 3 Institut de Soins Palliatifs et de Fin de Vie Michel-Sarrazin—Université Laval, Quebec, QC G1V 0A6, Canada; 4 Réseau Québécois de Recherche en Soins Palliatifs et de Fin de Vie (RQSPAL), Quebec, QC G1J 1Z4, Canada; 5 Faculty of Medicine, Université Laval, Quebec, QC G1V 0A6, Canada; 6 Population Health and Optimal Health Practices Program, CHU de Québec-Université Laval Research Center, Quebec, QC G1S 4L8, Canada; elodie.benard@crchudequebec.ulaval.ca (E.B.); alexandra.chapdelaine.1@ulaval.ca (A.C.); jason.guertin@fmed.ulaval.ca (J.R.G.); 7 Department of Social and Preventive Medicine, Faculty of Medicine, Université Laval, Quebec, QC G1V 0A6, Canada; 8 Department of Psychiatry and Addictology, Faculty of Medicine, Université de Montréal, Montreal, QC H3T 1J4, Canada; nicolas.garel@umontreal.ca; 9 Centre Hospitalier de l’Université de Montréal Research Center (CRCHUM), Montreal, QC H2X 0A9, Canada

**Keywords:** psilocybin-assisted therapy, existential distress, palliative care, implementation, qualitative analysis, cost analysis

## Abstract

**Background/Objectives**: Psilocybin-assisted therapy (PAT) is a promising intervention to alleviate existential distress among patients with advanced cancer receiving palliative care. However, evidence on how to integrate PAT into routine oncology and palliative care services remains scarce. This study aimed to examine real-world PAT implementation, identify factors influencing adoption, and estimate integration costs within oncology and palliative care services. **Methods**: We conducted a single-case implementation study in a large university-affiliated tertiary care center in Canada during the first year following its introduction. Semi-structured interviews with clinicians, managers, and other stakeholders explored barriers, facilitating conditions, and actions needed to support PAT implementation. A budget impact analysis estimated incremental costs associated with delivering PAT. **Results:** After one year, no patients had received PAT. Ten professionals representing diverse clinical and managerial roles participated in the interviews. While participants viewed PAT favorably, they emphasized the need to align the intervention with existing care pathways and clarify referral processes. Administrative and regulatory procedures, together with logistical constraints related to treatment delivery, were identified as key barriers, whereas perceived clinical relevance and institutional leadership were seen as important facilitators. From the health care system perspective, the estimated cost of delivering a complete PAT intervention ranged from 2648 to 5827 Canadian dollars (CAD) per patient, depending on the scenario examined, excluding the cost of the psilocybin itself. **Conclusions**: Despite perceived clinical relevance and relatively modest estimated costs, the absence of treated patients after one year highlights the gap between regulatory authorization and effective service uptake. These findings underscore the importance of structured implementation strategies, sustained institutional support, and alignment between regulatory frameworks and clinical workflows to ensure meaningful integration of PAT into routine oncology and palliative care services.

## 1. Introduction

Psilocybin is the psychoactive compound found in certain species of mushrooms. In clinical settings, psilocybin-assisted therapy (PAT) typically involves a structured therapeutic process combining preparatory psychotherapy, administration of a psychoactive dose of psilocybin in a supervised and supportive environment, and post-session integration psychotherapy aimed at helping patients process and integrate the experience into their broader psychological and existential context. PAT has gained increasing international attention in palliative care, where unmet needs related to existential distress and psychological suffering remain substantial [1,2]. Although psilocybin continues to be classified as a controlled substance in most jurisdictions, several countries have introduced regulated pathways allowing its therapeutic use [3]. Access to PAT is generally framed as an exceptional and a tightly regulated intervention rather than a standard pharmacological treatment, resulting in heterogeneous regulatory frameworks and clinical delivery models across jurisdictions. In addition to Australia, Switzerland, and selected U.S. states, Canada is among the early adopters to have integrated PAT into formal clinical or regulatory frameworks [3,4], while countries such as Germany, New Zealand, and the Czech Republic have more recently introduced similar regulatory pathways [5,6].

Evidence supporting PAT has largely been derived from small randomized controlled trials conducted among patients with life-threatening cancer [7,8,9]. These studies demonstrated rapid and sustained reductions in anxiety and depression in advanced cancer patients following a single supervised psilocybin session combined with psychotherapy. Follow-up and observational studies further suggest that these benefits may persist for months or even years in a substantial proportion of patients [10].

While clinical trials establish the therapeutic promise of PAT, they provide limited insight into how such outcomes can be achieved when the intervention is implemented within routine oncology and palliative care services. A growing body of literature highlights persistent implementation challenges, including the need for specialized clinician training, alignment with into existing clinical workflows, navigation of evolving regulatory frameworks, and sustained organizational support [11,12,13].

Beyond clinical and organizational considerations, the resources required to deliver PAT may also influence its adoption in routine care. Although the therapeutic model typically involves a limited number of sessions, PAT requires extended clinician involvement, specialized training, and dedicated treatment environments [14,15,16,17]. However, empirical data on the economic implications of integrating PAT into clinical practice remain scarce and have primarily focused on the treatment of depressive disorders rather than palliative care [18,19]. Estimating the resources required to deliver PAT in real-world settings may therefore help inform institutional planning and policy decisions regarding the feasibility and sustainability of this emerging intervention.

In Canada, the regulatory environment has evolved rapidly. Since January 2022, the Special Access Program has allowed health care providers to request restricted access to psilocybin for patients with end-of-life distress or major depressive disorder who have not responded to conventional treatments [20]. Health Canada’s Special Access Program allows healthcare practitioners to request access to drugs that have not been approved for general sale in Canada. These medications are typically still in development or have not yet completed the formal regulatory review required for market authorization. Access is granted on a case-by-case basis under Health Canada’s discretionary authority, without predefined thresholds for evidentiary sufficiency regarding efficacy. Similar regulatory pathways exist internationally, including the U.S. Food and Drug Administration’s Expanded Access and “Right to Try” programs. In December 2022, Quebec became the first Canadian province to approve public reimbursement for PAT through its health insurance system [21]. Together, these developments have created new opportunities for clinical innovation while simultaneously introducing organizational and procedural constraints that shape how PAT can be delivered in practice. This pilot study aimed to examine the real-world implementation of PAT within oncology and palliative care services in a large university-affiliated tertiary care center in Canada during the first year following its introduction. The specific objectives were to:

- Identify barriers and facilitators influencing implementation from the perspectives of stakeholders across involved departments and services.
- Estimate the costs associated with implementing PAT within this setting from both the hospital and broader health care system perspectives.

## 2. Materials and Methods

### 2.1. Study Design

This research was conducted alongside an ongoing clinical and organizational initiative but remained methodologically independent from clinical care delivery. Given the study’s aim to understand how PAT was operationalized in a real-world care context and to assess the potential transferability of the model developed at the study site to other settings, a single-case study design was selected.

### 2.2. Conceptual Frameworks

Two complementary conceptual frameworks guided the methodological choices and analytic approach. The first is Rogers’ Diffusion of Innovations theory, which posits that adoption is facilitated when an innovation (here PAT) is perceived as advantageous, compatible with existing practices, manageable in complexity, testable on a small scale, producing observable benefits, and adaptable to local contexts [22]. The second is the sociopolitical evaluation model of health innovations developed by Lehoux and Blume [23], which highlights how the adoption and implementation of technological, organizational, and therapeutic innovations are shaped by their social and political context, including the influence of key actors, available knowledge, resources, and power relations within health systems [23]. Together, these frameworks informed the analysis of organizational determinants of PAT adoption and the broader contextual dynamics shaping its integration into oncology and palliative care services.

### 2.3. Case Setting

The study was conducted in a large university-affiliated tertiary care center in Canada, within a universally publicly funded health system, part of an integrated academic health network associated with a major medical school. The institution comprises multiple hospital sites and provides specialized and highly specialized services to a population of more than two million people across a large geographic region. Oncology is a major clinical and academic focus of the institution. Its cancer program delivers services across the full continuum of care—from diagnosis and active treatment to survivorship and palliative care—through multidisciplinary teams including medical oncology, radiation oncology, surgery, pharmacy, and supportive care services. A large integrated cancer center located on one of the hospital campuses centralizes much of the outpatient oncology activity and supports both clinical care and research. Within this context, as part of a clinician-led initiative, PAT was introduced in January 2025 as a new clinical service within psycho-oncology and palliative care and was presented to clinicians across the department during an institutional presentation. As psilocybin remains a controlled substance in Canada, specific administrative steps are required to implement PAT. In particular, prior approval from an institutional pharmacy committee is needed before applying to the federal health authority’s Special Access Program, which, under strict regulations, allows healthcare professionals to access drugs or medical devices not yet approved for sale in Canada.

### 2.4. Data Collection

#### 2.4.1. Stakeholders’ Perspectives

Participants were purposively sampled to capture diverse perspectives among professionals involved in oncology and palliative care services. Eligible participants included clinicians, managers, and other stakeholders with experience in oncology and/or palliative care services and familiarity with the implementation context under study. Patients and caregivers were excluded from the sampling strategy because the study focused specifically on organizational and professional perspectives related to implementation. Personalized email invitations were sent to individuals identified by the research team as being involved in these services. Detailed participant characteristics are presented in Section 3.1. Personalized email invitations were sent to clinicians, managers, and other stakeholders identified by the research team as being involved in oncology and palliative care services. This approach aimed to recruit participants representing different roles across the care pathway who could provide informed perspectives on the introduction of PAT within the institution [24]. Individual semi-structured interviews, lasting between 30 and 60 min, were conducted by one author (M.M.-G.) between September 2025 and February 2026 with professionals working in oncology, palliative and supportive care, and pharmacy services. The interviewer had no prior relationship with participants and was not previously known to them. Interviews were conducted either in person or via Microsoft Teams, depending on participants’ availability and preferences. With participants’ consent, interviews were audio-recorded. The semi-structured interview guide was collaboratively developed by the research team based on the study objectives, the existing implementation literature, and the conceptual frameworks guiding the study. Questions explored participants’ perceptions regarding the integration of PAT into existing care pathways, perceived barriers and facilitating conditions, and actions needed to support implementation. Example questions included: “How do you perceive the integration of PAT within existing oncology and palliative care services?” and “What organizational or practical challenges do you foresee regarding the implementation of PAT in this setting?” The complete interview guide is provided in Supplementary Material File S1.

#### 2.4.2. Cost Estimates

Documentary analysis of internal policies, clinical protocols, and administrative records was conducted to map the PAT care pathway within the institution and to identify the resources required for service delivery at each stage of care. Costs relevant to both the hospital and health care system perspectives were considered and derived from publicly available remuneration schedules and institutional data. These included physician fees, salary costs for other health professionals involved in care delivery (e.g., nurses and psychologists), and overhead costs associated with the use of clinical space. In this analysis, the cost of psilocybin was assumed to be zero, as the psilocybin used for PAT within the publicly funded health care system was provided free of charge to clinicians by the pharmaceutical supplier at the time of the study. All costs are reported in 2025 Canadian dollars (CAD). For reference, 1 CAD ≈ 0.73 USD and ≈0.62 EUR at the time of writing.

### 2.5. Data Analysis

#### 2.5.1. Qualitative Analysis

All interviews were transcribed verbatim. The research team has prior training and experience in qualitative data analysis. Data were analyzed using a reflexive thematic analysis approach [25] supported by the qualitative data analysis software Delve (cloud-based version; Delve, Brisbane, Australia). Following Braun and Clarke’s six-step process [26], analysis was conducted in an iterative and non-linear manner. The analysis combined deductive and inductive processes, whereby the interview guide informed the overarching analytic domains, while themes and subthemes within these domains were developed reflexively and iteratively from the data. One author (M.M.-G.) conducted the initial coding and thematic synthesis based on the interview guide, interview transcripts and analytic notes. To support interpretation and reduce the risk of overly idiosyncratic interpretations, emerging codes and themes were discussed throughout the analytic process with the first author (M.D.) and later reviewed within the research team. These discussions helped refine the thematic organization and strengthen analytic credibility. This qualitative aspect of the study is reported according to the Consolidated Criteria for Reporting Qualitative Research (COREQ) guidelines (see Supplementary Material File S2).

#### 2.5.2. Cost of the Hypothetical PAT Health Care Pathway

Implementation costs and anticipated impacts on health care utilization were examined through an exploratory budget impact analysis combining cost data related to PAT delivery with projected resource use. To support this analysis, we developed a cost-calculation tool using Microsoft Excel 365 (Microsoft Corporation, Redmond, WA, USA) to estimate potential costs associated with PAT across each stage of the care pathway. The tool incorporates a set of assumptions regarding the types of health professionals involved, their remuneration, and the duration and format of the interventions delivered. Cost estimates were generated from two perspectives: that of the health care system and that of the hospital. From the health care system perspective, costs included physician and other health professional fees, and overhead expenses related to service delivery, such as facility operations and maintenance of the treatment space. From the hospital perspective, the same cost components were considered, with the exception of physician fees, which are covered publicly within Canada’s universal health care system. Accordingly, hospital-level costs included salaries of non-physician health professionals, psilocybin, and overhead costs associated with service delivery.

### 2.6. Ethical Considerations

This study was approved by the relevant institutional ethics committee. To protect participant confidentiality, the name of the institution is omitted (Ref: 2025-7809). Written informed consent was obtained from all participants. Data were collected anonymously and used solely for research purposes. Results were aggregated, ensuring no participant could be identified.

## 3. Results

**Figure 1:**
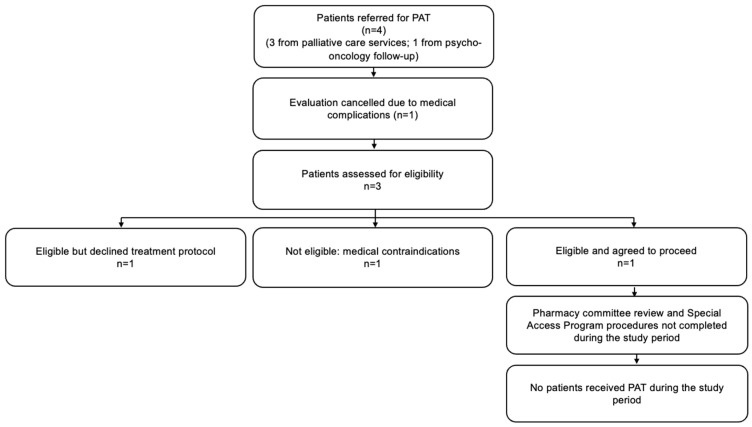
Flow of patients referred for PAT during the study period.

After one year of implementation, no patients had received PAT (Figure 1). Four patients were referred, primarily from palliative care services, for existential distress associated with advanced cancer. One patient cancelled the evaluation because of acute medical complications. Three patients underwent eligibility assessment. One was found medically ineligible because of significant hepatic dysfunction, another declined treatment after learning more about the institutional treatment protocol and conditions of administration, and one was assessed as eligible and agreed to proceed. However, progression toward treatment required institutional pharmacy committee review and subsequent Special Access Program procedures, which were not completed during the study period due to the broader administrative and organizational challenges associated with implementing this novel intervention.

### 3.1. Stakeholders’ Perspectives

Fourteen professionals involved in oncology and palliative care services within the institution were contacted. Two were excluded due to insufficient knowledge of the implementation of PAT, one had moved to a different role, and one referred a colleague to take their place. The final sample comprised 10 participants. Most were women (8/10, 80%), and the majority were between 41 and 60 years of age (9/10, 90%). Participants generally had extensive experience working within the institution, with most having worked there for 15 years or more (8/10, 80%). Participants represented a range of professional roles, including psychologists, psychiatrists, pharmacists, clinical support staff, coordinators, and managers. To preserve confidentiality, the number of participants within each professional category is not reported. Their roles ranged from direct patient care to service coordination and program oversight, and several were also involved in research or innovation initiatives within the institution. Together, these varied roles provided complementary perspectives on the clinical, organizational, and administrative dimensions involved in introducing PAT within the institution.

#### 3.1.1. Integration of PAT Within Existing Care Pathways

While there was general openness toward PAT, respondents emphasized that successful implementation would require alignment with established clinical processes. The current strategy of introducing PAT gradually with a small number of patients was generally perceived as reassuring, as it limits short-term pressure on financial and human resources.

*“In fact, what is somewhat reassuring is that it’s still a small volume. It’s a volume that we’re able to plan over time. So, I think it will require a slightly different organization of work once we identify patients who could benefit from PAT. But I don’t see any major obstacles in terms of work organization for psychotherapists.”*

(P4)

Even with a gradual integration of PAT, some participants expressed concern that once PAT becomes more formally integrated into routine care, increased demand could place additional pressure on already constrained clinical resources. However, some participants noted that this approach may also slow the transition toward a more structured and sustainable service model. Several respondents stressed that beginning to treat patients with PAT is essential to progress, as the absence of clinical activity may hinder institutional momentum. Participants also emphasized the importance of clarifying professional roles and establishing clear referral mechanisms to identify eligible patients. Several noted that without well-defined responsibilities and referral pathways, integrating PAT into routine clinical practice could remain challenging.

*“Well, who prescribes this medication? Because it’s still considered, I’d say, kind of like a drug, maybe. Who prescribes it? Who has access to it? Are we using it properly? I don’t really know how it works. Do pharmacists need to be involved in this?… Is it mostly psychiatrists who do this? Do they write prescriptions? Is there an established protocol? You know, I imagine… it’s not something that applies to everyone… Is it only done at [name of the institution]? Or is it being implemented across the province?”*

(P6)

#### 3.1.2. Perceived Barriers and Challenges

Administrative and regulatory procedures were widely perceived as major barriers to implementation. Many participants described documentation requirements and approval processes as burdensome and potentially discouraging for clinicians seeking to initiate treatment. In particular, the completion of the documentation required for the Special Access Program was frequently mentioned as a significant obstacle and a substantial administrative burden. Some participants also highlighted that the hospital environment requires multiple layers of internal authorization, including review by the institutional pharmacy committee. According to those respondents, these approval processes may represent a substantial hurdle, as their evaluation criteria often rely on evidence from large phase III clinical trials. Such evidence is rarely available in palliative care settings, notably with respect to PAT, where the literature more often consists of small randomized trials, observational studies, and case reports.

*“It’s really about freeing up time for people who want to work on this and bring it forward. I’m convinced we won’t have trouble finding refractory patients. There won’t be 50 a year, but I think it’s just… I see it more as an administrative process that’s a barrier, more than the teams on the ground. I don’t think there are prejudices anymore—that time is over. People aren’t saying, “Oh no, that’s weird, I don’t want to give that.” I think people want to try new things, they want to be equipped to do it, but the institution also needs to get behind us. In the end, they’re the ones who have the final say.”*

(P10)

Beyond administrative constraints, some respondents also pointed to practical challenges associated with the organization of PAT sessions. These included the length of treatment sessions, the need for the involvement of multiple professionals, and the requirement for a suitable therapeutic environment. Such constraints were perceived as difficult to accommodate within existing clinical workloads and available resources should this intervention expand.

#### 3.1.3. Facilitating Conditions

Several participants emphasized the clinical relevance of PAT for addressing existential distress among patients with advanced cancer, an aspect of suffering often insufficiently addressed by conventional treatments. As one participant summarized succinctly, “*the need is there*”.

Strong clinical leadership and the credibility of the clinicians and researchers involved in the institutional PAT initiative were also viewed as important facilitating factors. Respondents suggested that the support of respected leaders within the institution could help legitimize the intervention and foster broader institutional engagement. Some also highlighted that the university-affiliated environment of the institution created favorable conditions for exploring emerging therapeutic approaches. The organization’s commitment to research and innovation was perceived as supportive of initiatives aimed at developing new models of care.

*“Well, I think it’s more about… a kind of harmony, more and more. At [name of the institution], we want to be humane, we want to provide the best care, and we have a mission of research and innovation. So all of these missions fit really well with this kind of innovative project… And as a patient, or as a family member, I would feel very reassured knowing that these kinds of innovative therapies are being offered in a university-based center rather than in some X, Y, Z clinic. That would be reassuring to me.”*

(P5)

#### 3.1.4. Actions Needed to Support Successful Implementation

Participants identified several actions that could facilitate the successful implementation of PAT. These included developing training opportunities for professionals involved in delivering the intervention and generating evidence on both the clinical benefits and economic implications of PAT. Such data were viewed as essential to demonstrate the added value of the intervention and support its potential integration into routine clinical practice. Improving communication across clinical teams regarding the objectives, processes, and current status of PAT was also frequently mentioned as a priority. Participants suggested that clearer communication and coordination between departments could help facilitate referrals and strengthen collaboration among the professionals involved.

*“Yeah, I think it’s really about documenting what we’ve done and the impacts. Like case studies, publications, everything we can produce. There’s the scientific side, but also the clinical impacts on patients, and then the structures, protocols, training needs, you know. In the end, it’s about convincing the right people. (…) I think it will come down to demonstrating clinical effects. If we see clear benefits for patients, then as clinicians, we tend to see it as a priority.”*

(P6)

### 3.2. Costs of PAT Implementation

To account for uncertainty regarding how PAT might be delivered in routine practice, two scenarios were examined (Table 1). The minimum-cost scenario assumed that key clinical activities were delivered by the least costly professionals and at the lower bound of estimated durations. In this scenario, the initial medical assessment was conducted by a family physician, preparatory psychotherapy lasted two hours and was delivered by a psychologist, the psilocybin administration session involved a family physician and a psychologist, and integration psychotherapy consisted of six hours of follow-up sessions delivered by a psychologist. In contrast, the maximum-cost scenario assumed that the costliest professionals were involved and that intervention durations corresponded to the upper bound of the estimated ranges. Under this scenario, the initial assessment was conducted by a psychiatrist, preparatory psychotherapy lasted four hours and was delivered by a psychiatrist, the psilocybin administration session involved both a family physician and a psychiatrist, and integration psychotherapy consisted of eight hours of follow-up sessions delivered by a psychiatrist.

From the health care system perspective, the estimated cost of delivering a complete PAT intervention ranged from CAD 2648 to CAD 5827 per patient, depending on the scenario examined (Table 2). These estimates included professional fees for physicians and other health professionals, and overhead costs associated with service delivery. From the hospital perspective, estimated costs ranged from CAD 23 to CAD 1206 per patient. The difference between the two perspectives is largely explained by physician remuneration, which is covered through the publicly funded health care system rather than through hospital budgets. As a result, hospital-level costs primarily reflected salaries of non-physician health professionals, and overhead expenses related to service delivery. Annual overhead costs associated with the clinical space used for PAT were estimated at approximately CAD 1889 per year, including expenses related to facility operations, maintenance, hygiene, and security. These costs are expected to be distributed across the number of patients treated annually and may vary depending on the extent to which the treatment room is used for PAT relative to other clinical activities.

## 4. Discussion

This study provides one of the first descriptions of the real-world introduction of PAT within oncology and palliative care services. Despite generally favorable perceptions among stakeholders and relatively modest estimated costs of delivery, no patients ultimately received PAT during the first year following the program’s introduction.

Rather than reflecting a lack of clinical interest or perceived relevance, this absence of treated patients appeared to be associated with the early and evolving nature of implementation processes within the institution. Clinical procedures, organizational structures, and regulatory workflows were still being developed concurrently with attempts to initiate care delivery.

While clinical trials have demonstrated promising effects of PAT on anxiety and depression among patients with advanced cancer, far less is known about how such interventions can be translated into routine clinical practice. Our findings highlight the gap that may emerge between organizational, regulatory authorization, and the effective delivery of innovative therapies in real-world care settings.

### 4.1. Early Implementation Challenges in PAT Integration

Several factors may help explain the limited uptake observed during the first year of implementation. As reported by some of our participants, introducing PAT requires coordination across multiple clinical and administrative processes, including patient identification, psychiatric assessment, regulatory authorization, and the organization of extended treatment sessions. In addition, clinicians must navigate evolving regulatory frameworks and ensure appropriate training and institutional support. Even when the perceived clinical value of an intervention is high, the complexity of integrating a novel therapy into existing care pathways may slow its adoption, particularly during early implementation phases [27,28]. These findings suggest that the transition from regulatory authorization to routine clinical implementation may be substantially more complex and resource-intensive than is sometimes assumed in discussions surrounding psychedelic-assisted therapies. In this context, implementation readiness may depend not only on clinical expertise and regulatory access but also on the gradual development of institutional procedures, interdisciplinary coordination, and operational support structures. Our observations are consistent with broader implementation research showing that the successful introduction of innovative therapies depends not only on clinical evidence but also on organizational readiness, workflow integration, and professional engagement [28,29].

### 4.2. Economic and Organizational Considerations

The economic analysis suggests that the direct costs of delivering PAT are primarily driven by clinician time during preparation, administration, and integration phases. In the present study, the cost of psilocybin itself was considered null, as the substance was provided free of charge by a pharmaceutical company through the Special Access Program. However, the cost of the substance is increasingly no longer covered by companies. Based on prices charged in clinical trials, the cost could reasonably range between CAD 500 and CAD 1000. While this is not negligible, it would still represent only a fraction of the total cost of treatment, suggesting that financial considerations alone are unlikely to constitute a major barrier to implementation, particularly in publicly funded health systems where physician remuneration is covered externally. However, if patients were required to bear the cost of psilocybin, it could be highly dissuasive and may contribute to the persistence of an underground or grey market, which may not serve patients’ or providers’ best interests.

Despite growing interest in psychedelic-assisted therapies, economic evidence remains limited, particularly in real-world care settings [30]. Most existing analyses rely on modeling approaches or projections derived from clinical trial data rather than on observed costs in routine practice [18,19,31,32,33,34]. Preliminary findings suggest that PAT may be cost-effective for conditions such as treatment-resistant depression, given the potential durability of its effects after a limited number of sessions [10,11]. In palliative care contexts, where patients often face time constraints and complex care needs, there is a clear need for more robust empirical data on real-world costs to inform decisions regarding feasibility, sustainability, and integration into health care systems. Although our estimates do not capture downstream impacts such as changes in health care use, indirect costs, or broader patient and family benefits, they provide an initial basis for future economic evaluations of PAT in this context.

### 4.3. Alternative Psychedelic Models and Ketamine-Based Approaches

Considering the practical barriers encountered during the early implementation of PAT, other psychedelic interventions may warrant consideration. In particular, ketamine-assisted therapy has received increasing attention as a potential option for addressing depression, anxiety, and psychological distress among patients with advanced illness [35]. Unlike psilocybin, ketamine is an approved medication (e.g., as an anesthetic and included on the World Health Organization Essential Medicines List) and is routinely available through hospital pharmacies within established clinical frameworks. While its use in psychiatry is generally off-label, this accessibility may facilitate more rapid integration into clinical practice. Its established use in anesthesia and pain management, together with growing evidence supporting its rapid antidepressant effects, has led some clinicians to explore ketamine-based interventions as a means of alleviating severe psychological distress in patients with serious medical conditions [35].

However, ketamine-based interventions encompass distinct models of care that differ in their underlying mechanisms, expected durability of effects, and practical implications. In much of the psychiatric literature, ketamine is administered within a predominantly biomedical framework involving repeated dosing aimed at short-term symptom reduction [36]. Within this model, therapeutic effects are often transient and may require multiple administrations to maintain clinical benefit [37], which has implications for feasibility and costs in palliative care settings, particularly for patients with limited time, reduced mobility, or fluctuating clinical status. In contrast, emerging ketamine-assisted psychotherapy models place greater emphasis on the subjective and potentially transformative aspects of the acute experience, combined with psychotherapeutic support and integration [38,39]. Although still less well established, particularly in palliative populations, these approaches aim to achieve more sustained benefits with a limited number of sessions [39,40]. Recent psychometric and phenomenological findings further suggest that ketamine can occasion subjective experiences with features overlapping those observed with classic psychedelics [41], raising the possibility that, when embedded within an appropriate therapeutic framework, its effects may extend beyond short-lived symptom relief [41,42].

From a feasibility standpoint, comparisons between ketamine and psilocybin also warrant nuance. While ketamine protocols often involve multiple sessions, each session typically requires shorter periods of monitoring (about 2 h each) [43]. In practice, several ketamine sessions may represent a cumulative monitoring burden comparable to that of a single psilocybin session, which often requires prolonged continuous support (6–8 h) [44]. Taken together, while ketamine-based approaches may offer a pragmatic option in contexts where access to psilocybin remains restricted, important differences between treatment models should be carefully considered. Future research should examine how different psychedelic-assisted approaches, including both psilocybin and ketamine, can be integrated into palliative care in ways that balance clinical effectiveness, feasibility, and patient-centered considerations.

### 4.4. Implications for Practice and Research

Beyond the specific context of this case study, the findings contribute to the emerging implementation literature on psychedelic-assisted therapies by documenting the practical realities involved in translating PAT from research and regulatory frameworks into routine clinical care. Importantly, the absence of treated patients during the study period should not necessarily be interpreted as evidence of implementation failure, but rather as reflecting the complexity of introducing a novel and highly regulated intervention into existing health care structures during an early implementation phase. While the sample was relatively small and drawn from a single Canadian institution, participants occupied diverse clinical, managerial, and organizational roles directly involved in oncology and palliative care delivery. Their perspectives therefore provide insight into the institutional conditions that may influence the feasibility of PAT implementation in publicly funded health systems. The findings suggest that successful implementation depends not only on clinical evidence or regulatory authorization, but also on administrative feasibility, workflow integration, institutional leadership, and organizational readiness. These observations may help inform implementation efforts in other jurisdictions considering the integration of psychedelic-assisted therapies into clinical care.

More broadly, the study highlights the importance of complementing efficacy-focused psychedelic research with implementation and health systems perspectives. Future research should examine how different care models and regulatory frameworks influence the accessibility, sustainability, and scalability of PAT across diverse clinical settings.

### 4.5. Limitations

This study has several limitations. Given the exploratory nature of this service implementation study, findings should be interpreted within the specific context of a single tertiary care center and may not be fully generalizable to other settings. 

First, the qualitative component relied on a relatively small sample of professionals from a single institution, which may limit the transferability of the findings to other organizational or health system contexts. Although participants represented diverse clinical, managerial, and organizational roles relevant to PAT implementation, a larger sample may have captured additional perspectives and experiences. Participants were purposively recruited because of their involvement in palliative oncology care pathways, which may also have introduced perspectives more closely aligned with the institutional vision. In addition, interviews were conducted during an early phase of program development, when clinical activity had not yet begun, and participants’ views therefore reflected anticipated rather than fully observed implementation experiences.Moreover, the qualitative component focused exclusively on professional and organizational stakeholders involved in implementation processes and did not include patients, caregivers, or patient advocates. Given the highly patient-centered nature of existential distress and palliative care, the absence of these perspectives limits understanding of the acceptability and experiential dimensions of PAT implementation. Future research should incorporate patient and caregiver perspectives to better inform the development of accessible and patient-centered models of care.

Data saturation was not reached, and combined with the small sample size, this may have limited the diversity of perspectives captured. Although participants held heterogeneous roles, allowing documentation of multiple dimensions of implementation, this diversity may have contributed to a breadth rather than depth of insights within specific professional groups. Finally, the qualitative analysis was primarily conducted by a single researcher, consistent with a reflexive thematic analysis approach [25], in which coding is understood as an interpretive process rather than one aimed at inter-coder reliability. Although a formal reflexive journal was not maintained, interpretations were discussed iteratively with other members of the research team, supporting analytic rigor and the credibility of the findings. These methodological choices should be interpreted in light of the study’s early-stage implementation context, characterized by evolving organizational processes and the need to generate timely insights aligned with field constraints.

Second, the economic estimates were based on modeled scenarios and assumptions regarding professional involvement and intervention duration rather than observed clinical activity. Accordingly, these estimates should be interpreted as preliminary and primarily intended to inform future real-world economic evaluations of PAT implementation.

This analysis did not include the cost of the molecule itself and did not capture potential cost offsets or broader societal benefits associated with PAT. Future research should examine the longer-term implementation of PAT across multiple settings and assess both clinical outcomes and broader health system impacts. Prospective studies will be needed to better understand how regulatory frameworks, institutional processes, and clinical workflows influence the uptake of PAT in routine care.

## 5. Conclusions

This study highlights the gap that may emerge between therapeutic innovation and clinical implementation. While PAT is increasingly supported by clinical evidence and regulatory pathways, its integration into routine oncology and palliative care services remains complex. The absence of treated patients during the first year of deployment suggests that evidence and regulatory authorization alone may be insufficient to ensure meaningful access during early implementation phases. Understanding and addressing the organizational conditions required for implementation will be essential for translating the promise of PAT into real-world care.

## Figures and Tables

**Table 1:** Psilocybin-assisted therapy care pathway in the case study setting.

Phase of Care	Key Activities	Professionals Involved	Estimated Duration
Screening and eligibility	Initial review and information	Nurse	~0.5 h
Clinical assessment	Evaluation of indications and contraindications	Physician *	~2 h
Regulatory preparation	Preparation of regulatory request	Physician + pharmacist	~4 h
Preparatory psychotherapy	Psychological preparation	Physician or psychologist	~2–4 h
Psilocybin administration	Supervised dosing session	Physician + physician or psychologist	~8 h
Integration psychotherapy	Post-session integration	Physician or psychologist	~6–8 h

* Typically, the physician may be a family physician or a psychiatrist.

**Table 2:** Estimated costs of psilocybin-assisted therapy per patient in the case study setting *.

Perspective	Minimum Scenario	Maximum Scenario
Health care system *	CAD ** 2648	CAD 5827
Hospital ***	CAD 23	CAD 1206

* This analysis does not account for the cost of psilocybin, as it was provided free of charge by the pharmaceutical supplier at the time of the study. In routine practice, this cost would be expected to be included in the hospital perspective. ** Costs are expressed in Canadian dollars (CAD). For reference, 1 CAD ≈ 0.73 USD and ≈0.62 EUR at the time of writing; exchange rates may vary over time. *** Physician remuneration is covered by the publicly funded health care system and therefore excluded from hospital-level costs.

## Data Availability

Data supporting this study cannot be made available for ethical restrictions as it would compromise participant confidentiality.

## Supplementary Materials

The following supporting information can be downloaded at: https://www.mdpi.com/article/10.3390/healthcare14111559/s1, File S1: Interview Guide And Research Questionnaire; File S2: (COREQ): 32-item checklist. Reference [45] is cited in the Supplementary Materials.

## Abbreviations

The following abbreviations are used in this manuscript:

CAD	Canadian Dollars
COREQ	Consolidated Criteria for Reporting Qualitative Research
EUR	Euros
PAT	Psilocybin-Assisted Therapy
USD	United States Dollars

## References

[1] Lapid M.I., Pagali S.R., Randall A.L., Donovan K.A., Bronars C.A., Gauthier T.A., Bock J., Lim S.D., Carey E.C., Sokolowski E. (2025). Evaluating the effectiveness of psilocybin in alleviating distress among cancer patients: A systematic review. Palliat. Support. Care.

[2] Schuman H.D.M., Savard C., Mina R., Barkova S., Conradi H.S.W., Deleemans J.M., Carlson L.E. (2025). Psychedelic-Assisted Therapies for Psychosocial Symptoms in Cancer: A Systematic Review and Meta-Analysis. Curr. Oncol..

[3] (2026). Worldwide Psychedelic Laws. https://psychedelicalpha.com/resources/worldwide-psychedelic-laws/.

[4] Gouvernement du Canada (2022). Règlement Modifiant Certains Règlements Visant les Drogues d’Usage Restreint (Programme d’Accès Spécial).

[5] Madero S., Soto-Angona O., Ona G., Sanchez-Moreno J., Vieta E. (2026). Current perspectives on psychedelic treatments in Europe. Lancet Reg. Health—Eur..

[6] New Zealand Medicines and Medical Devices Safety Authority (2025). Applications for Approval to Prescribe Psychedelics for Psychedelic-Assisted Therapy. https://www.medsafe.govt.nz/profs/psychedelics.asp.

[7] Grob C.S., Danforth A.L., Chopra G.S., Hagerty M., McKay C.R., Halberstadt A.L., Greer G.R. (2011). Pilot Study of Psilocybin Treatment for Anxiety in Patients with Advanced-Stage Cancer. Arch. Gen. Psychiatry.

[8] Griffiths R.R., Johnson M.W., Carducci M.A., Umbricht A., Richards W.A., Richards B.D., Cosimano M.P., Klinedinst M.A. (2016). Psilocybin produces substantial and sustained decreases in depression and anxiety in patients with life-threatening cancer: A randomized double-blind trial. J. Psychopharmacol..

[9] Ross S., Bossis A., Guss J., Agin-Liebes G., Malone T., Cohen B., Mennenga S.E., Belser A., Kalliontzi K., Babb J. (2016). Rapid and sustained symptom reduction following psilocybin treatment for anxiety and depression in patients with life-threatening cancer: A randomized controlled trial. J. Psychopharmacol..

[10] Agin-Liebes G.I., Malone T., Yalch M.M., Mennenga S.E., Ponté K.L., Guss J., Bossis A.P., Grigsby J., Fischer S., Ross S. (2020). Long-term follow-up of psilocybin-assisted psychotherapy for psychiatric and existential distress in patients with life-threatening cancer. J. Psychopharmacol..

[11] Dorval M., Chang S.L., Farzin H., Nguyen O., Stephan J.F., Tapp D., Deschamps P., Joly Y., Moureaux F., Foxman R. (2025). Roadmap for Equitable Access and Responsible Use of Psilocybin-Assisted Psychotherapy in Palliative Care. Palliat. Med. Rep..

[12] Williams M.L., Korevaar D., Harvey R., Fitzgerald P.B., Liknaitzky P., O’Carroll S., Puspanathan P., Ross M., Strauss N., Bennett-Levy J. (2021). Translating Psychedelic Therapies from Clinical Trials to Community Clinics: Building Bridges and Addressing Potential Challenges Ahead. Front. Psychiatry.

[13] Wolff M., Rutrecht H., Gründer G., Jungaberle A., Jungaberle H. (2025). Key competencies for psychedelic treatment in real-world mental health care settings. Gen. Hosp. Psychiatry.

[14] Estric C., Duron T., Kabani S., Lopez-Castroman J. (2025). Set and setting of psychedelics for therapeutic use in psychiatry: A systematic review. J. Psychopharmacol..

[15] Bhatt K.V., Asuncion J.D., Alam A., Zisook S., Stahl S.M. (2025). Should we skip the trip? Clinical implications of psychedelic-associated subjective effects and the potential role of non-hallucinogenic alternatives. Gen. Hosp. Psychiatry.

[16] Horton D.M., Morrison B., Schmidt J. (2021). Systematized Review of Psychotherapeutic Components of Psilocybin-Assisted Psychotherapy. Am. J. Psychother..

[17] Morland L., Woolley J.U.S., Department of Veteran Affairs (2025). PTSD: National Center for PTSD. Psychedelic-Assisted Therapy for PTSD. https://www.ptsd.va.gov/professional/treat/txessentials/psychedelics_assisted_therapy.asp.

[18] Avanceña A.L.V., Vuong L., Kahn J.G., Marseille E. (2025). Psilocybin-assisted therapy for treatment-resistant depression in the US: A model-based cost-effectiveness analysis. Transl. Psychiatry.

[19] McCrone P., Fisher H., Knight C., Harding R., Schlag A.K., Nutt D.J., Neill J.C. (2023). Cost-effectiveness of psilocybin-assisted therapy for severe depression: Exploratory findings from a decision analytic model. Psychol. Med..

[20] Garel N., Plourde L., Greenway K.T., Dorval M. (2025). The promise and challenges of psychedelic-assisted therapy: Lessons from Canada’s Special Access Program. Nat. Ment. Health.

[21] Herrington A. (2022). Quebec Approves Health Coverage for Psilocybin Therapy. Forbes Magazine. https://www.forbes.com/sites/ajherrington/2022/12/16/quebec-approves-health-coverage-for-psilocybin-therapy/?sh=73a88cae7fa9.

[22] Rogers E. (2003). Diffusion of Innovations.

[23] Lehoux P., Blume S. (2000). Technology Assessment and the Sociopolitics of Health Technologies. J. Health Politics Policy Law.

[24] Palinkas L.A., Horwitz S.M., Green C.A., Wisdom J.P., Duan N., Hoagwood K. (2015). Purposeful sampling for qualitative data collection and analysis in mixed method implementation research. Adm. Policy Ment. Health Ment. Health Serv. Res..

[25] Braun V., Clarke V. (2021). Thematic Analysis: A Practical Guide.

[26] Braun V., Clarke V. (2012). Thematic analysis. APA Handbook of Research Methods in Psychology, Vol 2: Research Designs: Quantitative, Qualitative, Neuropsychological, and Biological.

[27] Greenhalgh T., Robert G., Macfarlane F., Bate P., Kyriakidou O. (2004). Diffusion of innovations in service organizations: Systematic review and recommendations. Milbank Q..

[28] Damschroder L.J., Aron D.C., Keith R.E., Kirsh S.R., Alexander J.A., Lowery J.C. (2009). Fostering implementation of health services research findings into practice: A consolidated framework for advancing implementation science. Implement. Sci..

[29] Weiner B.J. (2009). A theory of organizational readiness for change. Implement. Sci..

[30] Marseille E., Bertozzi S., Kahn J.G. (2022). The economics of psychedelic-assisted therapies: A research agenda. Front. Psychiatry.

[31] Ziadi Y., Park T. (2026). Cost-Effectiveness of Psilocybin-Assisted Therapy Versus Standard of Care for Patients with Treatment-Resistant Depression. Value Health Reg. Issues.

[32] Marseille E., Kahn J.G., Yazar-Klosinski B., Doblin R. (2020). The cost-effectiveness of MDMA-assisted psychotherapy for the treatment of chronic, treatment-resistant PTSD. PLoS ONE.

[33] Marseille E., Mitchell J.M., Kahn J.G. (2022). Updated cost-effectiveness of MDMA-assisted therapy for the treatment of posttraumatic stress disorder in the United States: Findings from a phase 3 trial. PLoS ONE.

[34] Avanceña A.L.V., Kahn J.G., Marseille E. (2022). The Costs and Health Benefits of Expanded Access to MDMA-assisted Therapy for Chronic and Severe PTSD in the USA: A Modeling Study. Clin. Drug Investig..

[35] Sholevar R., Kromka W., Beaussant Y. (2025). Ketamine and Ketamine-Assisted Psychotherapy for Psychiatric and Existential Distress in Patients with Serious Medical Illness: A Narrative Review. J. Palliat. Med..

[36] Kohtala S. (2021). Ketamine-50 years in use: From anesthesia to rapid antidepressant effects and neurobiological mechanisms. Pharmacol. Rep..

[37] Dean R.L., Hurducas C., Hawton K., Spyridi S., Cowen P.J., Hollingsworth S., Marquardt T., Barnes A., Smith R., McShane R. (2021). Ketamine and other glutamate receptor modulators for depression in adults with unipolar major depressive disorder. Cochrane Database Syst. Rev..

[38] Campolina A.G., de Oliveira M.A.T. (2025). Unfolding States of Mind: A Dissociative-Psychedelic Model of Ketamine-Assisted Psychotherapy in Palliative Care. Healthcare.

[39] Garel N., Drury J., Thibault Lévesque J., Goyette N., Lehmann A., Looper K., Erritzoe D., Dames S., Turecki G., Rej S. (2023). The Montreal model: An integrative biomedical-psychedelic approach to ketamine for severe treatment-resistant depression. Front. Psychiatry.

[40] Diep D., de la Salle S., Thibault Lévesque J., Lifshitz M., Garel N., Greenway K.T. (2025). The ketamine chameleon: History, pharmacology, and the contested value of experience. Expert Rev. Clin. Pharmacol..

[41] Greenway K.T., Garel N., Dinh-Williams L.-A.L., Thibault Lévesque J., Kaelen M., Dagenais-Beaulé V., de la Salle S., Erritzoe D., Looper K., Turecki G. (2026). The Music for Subanesthetic Infusions of Ketamine randomised clinical trial: Ketamine as a psychedelic treatment for highly refractory depression. Br. J. Psychiatry.

[42] Garel N., Greenway K.T., Dinh-Williams L.-A.L., Thibault-Levesque J., Jutras-Aswad D., Turecki G., Rej S., Richard-Devantoy S. (2023). Intravenous ketamine for benzodiazepine deprescription and withdrawal management in treatment-resistant depression: A preliminary report. Neuropsychopharmacology.

[43] Swainson J., McGirr A., Blier P., Brietzke E., Richard-Devantoy S., Ravindran N., Blier N., Beaulieu S., Frey B.N., Kennedy S.H. (2021). The Canadian Network for Mood and Anxiety Treatments (CANMAT) Task Force Recommendations for the Use of Racemic Ketamine in Adults with Major Depressive Disorder. Can. J. Psychiatry.

[44] MacCallum C.A., Lo L.A., Pistawka C.A., Deol J.K. (2022). Therapeutic use of psilocybin: Practical considerations for dosing and administration. Front. Psychiatry.

[45] Tong A., Sainsbury P., Craig J. (2007). Consolidated criteria for reporting qualitative research (COREQ): A 32-item checklist for interviews and focus groups. Int. J. Qual. Health Care.

