# Supplementary material for: Bringing Psilocybin-Assisted Therapy to Palliative Oncology: Early Lessons from Real-World Implementation
Source: Healthcare (Basel). 2026 Jun 3;14(11):1559. doi: 10.3390/healthcare14111559 (PMC13257048; doi:10.3390/healthcare14111559)
Supplement: Supplementary file 1 [file healthcare-14-01559-s001.zip › healthcare-4310057-supplementary S2.pdf]

(COREQ): 32-item checklist

Developed from:

Tong A, Sainsbury P, Craig J. Consolidated criteria for reporting qualitative research (COREQ): a 32-item checklist for interviews and focus groups. *International Journal for Quality in Health Care*. 2007. Volume 19, Number 6: pp. 349 – 357

**No. Item**	**Guide questions/description**	**Reported on Page ***
**Domain 1: Research team and reflexivity**		
Personal Characteristics		
1. Interviewer/facilitator	Which author/s conducted the interview or focus group?	Page 4
2. Credentials	What were the researcher's credentials? E.g. PhD, MD	Not reported as not required by the journal on the title page: *MSc, PhD*
3. Occupation	What was their occupation at the time of the study?	Not reported as not required by the journal on the title page: *The authors who were involved in the qualitative research were researcher and research professional.*
4. Gender	Was the researcher male or female?	Not reported in the text: *Male and female*
5. Experience and training	What experience or training did the researcher have?	Page 4
Relationship with participants		
6. Relationship established	Was a relationship established prior to study commencement?	Page 4
7. Participant knowledge of the interviewer	What did the participants know about the researcher? e.g. personal goals, reasons for doing the research	Not reported in the text: *Ethical requirements required the*

		interviewer to describe the project, its objectives, and to answer the questions posed in the invitation email or during the interview.
8. Interviewer characteristics	What characteristics were reported about the inter viewer/facilitator? e.g. Bias, assumptions, reasons and interests in the research topic	Not reported in the text: *The interviewer was trained and remained neutral on the subject.*<br>*The participants knew that the interviewer belonged to the P3A research group, which is overseeing the implementation project*
**Domain 2: study design**		
Theoretical framework		
9. Methodological orientation and Theory	What methodological orientation was stated to underpin the study? e.g. grounded theory, discourse analysis, ethnography, phenomenology, content analysis	Pages 4-5
Participant selection		
10. Sampling	How were participants selected? e.g. purposive, convenience, consecutive, snowball	Page 4
11. Method of approach	How were participants approached? e.g. face-to-face, telephone, mail, email	Page 4
12. Sample size	How many participants were in the study?	Page 5
13. Non-participation	How many people refused to participate or dropped out? Reasons?	Page 5
Setting		
14. Setting of data collection	Where was the data collected? e.g. home, clinic, workplace	Page 4

15. Presence of non-participants	Was anyone else present besides the participants and researchers?	No
16. Description of sample	What are the important characteristics of the sample? e.g. demographic data, date	Page 4-5
Data collection		
17. Interview guide	Were questions, prompts, guides provided by the authors? Was it pilot tested?	Page 4<br>*No, the interview guide has not been tested.*
18. Repeat interviews	Were repeat interviews carried out? If yes, how many?	Not applicable
19. Audio/visual recording	Did the research use audio or visual recording to collect the data?<br>.	Page 4
20. Field notes	Were field notes made during and/or after the interview or focus group?	No
21. Duration	What was the duration of the inter views or focus group?	Page 4
22. Data saturation	Was data saturation discussed?	Page 11
23. Transcripts returned	Were transcripts returned to participants for comment and/or correction?	No
**Domain 3: analysis and findings**		
Data analysis		
24. Number of data coders	How many data coders coded the data?	Page 4
25. Description of the coding tree	Did authors provide a description of the coding tree?	No
26. Derivation of themes	Were themes identified in advance or derived from the data?<br>.	Page 4-5
27. Software	What software, if applicable, was used to manage the data?	Page 4
28. Participant checking	Did participants provide feedback on the findings? *.*	No
Reporting		
29. Quotations presented	Were participant quotations presented to illustrate the themes/findings? Was each	Pages 6-7

	quotation identified? e.g. participant number<br>.	
30. Data and findings consistent	Was there consistency between the data presented and the findings?	Pages 6-7
31. Clarity of major themes	Were major themes clearly presented in the findings?	Pages 6-7
32. Clarity of minor themes	Is there a description of diverse cases or discussion of minor themes?	Pages 6-7<br>Secondary themes are discussed in a narrative manner.
* Page numbers refer to the manuscript as originally submitted to the journal		
