# Supplementary material for: Bringing Psilocybin-Assisted Therapy to Palliative Oncology: Early Lessons from Real-World Implementation
Source: Healthcare (Basel). 2026 Jun 3;14(11):1559. doi: 10.3390/healthcare14111559 (PMC13257048; doi:10.3390/healthcare14111559)
Supplement: Supplementary file 1 [file healthcare-14-01559-s001.zip › healthcare-4310057-supplementary S1.pdf]

INTERVIEW GUIDE AND RESEARCH QUESTIONNAIRE

Issues and challenges in implementing psilocybin-assisted therapy at [Name of the institution]: A case study

Participant Identification Number:
Interview Date:

IMPORTANT: No identifying or personally identifiable information about the respondent must be recorded on this questionnaire.

Interview Guidelines

Project Objective:

This research project aims to describe the challenges, opportunities, and outcomes associated with integrating psilocybin-assisted therapy (PAT) into oncology and palliative care services at [Name of the institution], with the goal of optimizing the delivery of this treatment in local and regional healthcare institutions across Quebec.

Practical Information and Instructions:

Before beginning the interview, we would like to remind you that there are no right or wrong answers. Our objective is to gather your comments and perceptions regarding the implementation of PAT within the institution.

A) Review the main points of the consent form with the participant:

- With your consent, the individual interview will be audio-recorded to facilitate data analysis;
- Your name will not appear in the transcripts, and your personal data, as well as your statements, will not be linked to your identity;
- Only coded and anonymized data may be shared by the principal researchers with other members of the research team;
- Your participation is voluntary. You are free to withdraw from the project at any time, including during the interview.

B) Ensure that no questions remain unanswered and that all information is understood by the participant before beginning the interview.

C) Remind the participant of the interview duration: a maximum of 45 minutes. Ensure that the participant has signed the consent form and completed the sociodemographic questionnaire before starting the individual interview.

Semi-Structured Individual Interview Guide

The discussion covers the following themes:

a) Theme 1: Integration of psilocybin-assisted therapy (PAT) into oncology and palliative care services

b) Theme 2: Issues and challenges

c) Theme 3: Facilitating conditions

d) Theme 4: Actions to prioritize for successful integration

Context:

Within the framework of this project, the integration of psilocybin-assisted therapy (PAT) refers to all aspects related to:

- Assessing a patient’s eligibility to receive PAT;
- The personnel involved in PAT;
- The prescription of psilocybin;
- The administration of psilocybin;
- The follow-up of individuals treated with PAT.

Start the Recording

At the beginning of the recording, state the following:

- “Individual interview # (participant’s assigned number)”
- The date of the interview
- The name of the institution
- Your name (the interviewer’s name)

Context Setting

- Could you first tell me about your role in relation to oncology and palliative care at [Name of the institution]?
- How did you learn that psilocybin-assisted therapy (PAT) was being implemented at [Name of the institution]?
- What were your initial reactions?

Theme 1: Integration of psilocybin-assisted therapy (PAT) into oncology and palliative care services

- In your view, what led the psychosocial and spiritual oncology team at [Name of the institution] to consider offering PAT to certain patients?
- How does this initiative align with the institution's mission?
- How do you see PAT being integrated into the current organization of oncology or palliative care services?
- How might your role at [Name of the institution] lead you to play a part in the implementation of PAT within the institution?

Theme 2: Issues and Challenges

- In your opinion, what are the current and future issues and challenges associated with integrating PAT within the institution?
- How could these be addressed or overcome? (Ask for concrete examples if necessary.)
- Do any of these issues and challenges still persist today? Please explain.

Theme 3: Facilitating Conditions

- In your opinion, what conditions can/could facilitate the integration of PAT within the institution?
- What role should the institution play in supporting the integration of PAT?

Theme 4: Priority Actions for Successful Integration

- In your opinion, what priority actions should be implemented to integrate PAT into the care offerings at [Name of the institution]?
- What actions should be implemented to assess the impact of PAT at [Name of the institution]?
- Do you have any additional comments you would like to share regarding the integration of PAT at [Name of the institution]?

Thank you for your participation and collaboration!
